# Effects of cigarette package colors and warning labels on marlboro smokers’ risk beliefs, product appraisals, and smoking behavior: a randomized trial

**DOI:** 10.1186/s12889-023-17024-5

**Published:** 2023-10-27

**Authors:** Matthew D. Stone, Melissa Mercincavage, E. Paul Wileyto, Andy S.L. Tan, Janet Audrain-McGovern, Andrea C. Villanti, Andrew A. Strasser

**Affiliations:** 1 Herbert Wertheim School of Public Health and Human Longevity Science, University of California San Diego, San Diego, CA USA; 2 Department of Psychiatry and Tobacco Center of Regulatory Science, Perelman School of Medicine, University of Pennsylvania, Philadelphia, PA USA; 3 Annenberg School for Communication, University of Pennsylvania, Philadelphia, PA USA; 4 Rutgers, School of Public Health, The State University of New Jersey, New Brunswick, NJ USA

**Keywords:** Cigarette packaging, Warning labels, Risk beliefs

## Abstract

**Objective:**

Plain packaging and graphic warning labels are two regulatory strategies that may impact cigarette risk beliefs and reduce consumption, but data are needed to better understand how smokers respond to such regulations.

**Methods:**

Adult, daily, Marlboro non-menthol smokers (Red [*n* = 141] or Gold [*n* = 43]) completed a mixed factorial randomized trial. Participants smoked their usual cigarettes during baseline (5-days) and were randomized to receive cigarette packs with a warning label manipulation (graphic vs. text-only). Within each warning label condition, participants completed three within-subjects pack color manipulations (red, gold, plain), each lasting 15 days. Participants were blinded to the fact that all packs contained their usual cigarettes. Mixed-effects models examined between- and within-subject differences on risk beliefs, product perceptions, and smoking behavior.

**Results:**

Warning type and package color did not impact cigarette consumption or subjective ratings. However, use increased in all conditions (2.59–3.59 cigarettes per day) relative to baseline. While smokers largely held correct risk beliefs at baseline (Mean = 6.02, SE = 0.17, Range:0–8), the cumulative number of incorrect or uncertain cigarette risk beliefs increased from baseline in all pack color manipulations in the text (IRR range = 1.70–2.16) and graphic (IRR range = 1.31–1.70) warning conditions. Across all pack color periods, those in the graphic (vs. text) warning condition had reduced odds of reporting their study cigarettes as ‘safer’ than regular cigarettes (OR range = 0.22–0.32).

**Conclusions:**

Pack color modification may increase uncertainty about several key cigarette risk beliefs, though graphic warnings may attenuate these effects. Regulatory agencies could consider supporting policy changes with information campaigns to maximize public knowledge.

**Trial registration:**

November 25, 2014; Registration number: NCT02301351.

**Supplementary Information:**

The online version contains supplementary material available at 10.1186/s12889-023-17024-5.

## Introduction

 While rates of combustible cigarette use in the United States (US) continue to decline [1] smoking still accounts for nearly half-a-million deaths annually [2] Graphic warning labels on cigarette packs depicting the health hazards of smoking have seen widespread global adoption (> 120 countries) and have effectively reduced tobacco use in other countries [3]. Yet, the US is one of the remaining countries without a graphic labeling requirement. After failed implementation of a 2011 graphic warning rule, [4] the Food and Drug Administration (FDA) recently finalized a new set of warnings to go into effect on October 6, 2023 [5]. These warnings aim to communicate smoking health risk information in a manner that helps the public understand the negative health consequences of use [6]. Recent US trials have shown that graphic warnings decreased smokers’ positive perceptions of their cigarettes [7, 8], and increase quitting-related cognitions [7, 9], quit attempts [9] and cessation after 4-weeks, [9] but not 3-months [7–9]. While graphic warnings appear to increase smoking-related health perceptions [7, 8], strong empirical data is needed to better understand changes in risk beliefs in response to graphic warning labels, particularly upon initial exposure.

With increasing regulations on cigarettes, packaging has been referred by tobacco companies as the “final communication vehicle” [10]. Cigarette packaging designs can affect smokers’ beliefs about brands, downplay the harms of smoking, and ultimately influence use behavior [10, 11]. Tobacco industry documents show that “the sensory experience of smoking a cigarette can be manipulated simply by changing the design elements of the pack, such as color, fonts and logos.” [12]. Industry research found that Marlboro Ultra-Light cigarettes are perceived to have a “harsher” drag when placed in red packs while cigarettes from blue packs were considered “too mild” [10, 12]. Yet, one of the most effective manipulation strategies employed by the industry was the introduction of ‘light’ cigarettes, which are not less addictive or lethal, but were packaged and marketed to imply ‘less harm.’ After FDA prohibited these misleading labels in 2010, many tobacco companies, guided by Philip Morris (manufacturer of Marlboro), shifted focus to use a cigarette pack color-coding scheme to imply that ‘light’ flavored cigarettes would be sold in gold packs while the ‘full flavor’ cigarettes would continue to be sold in red packs [13]. Removal of these misleading descriptors only led to a subtle change after the ban went into effect, with just a fraction of light smokers noticing modifications to their packs [14]. Nearly a decade after the ban, smokers continue to believe that gold cigarettes are not as harmful as their red counterparts, despite biological measures showing equivalent carcinogenic exposure [15]. To help combat these kinds of industry-generated misperceptions, Australia updated their graphic warning policy by enacting a Plain Packaging Law in 2011, which standardized pack color and prohibited any marketing materials from appearing in or on packs [16]. While 16 countries have followed suit and implemented similar policies, [17] a Plain Packaging policy is not currently under consideration in the US.

This study examined the effects of two cigarette packaging policies (warning label type and pack color) and their interaction on cigarette risk beliefs, product perceptions, and smoking behavior. We focused our attention on Marlboro cigarettes as they are the most popular domestic brand that is preferred by over 40% of US cigarette smokers [18]. We hypothesized that a graphic (vs. text) warning label condition would increase product risk perceptions and decrease subjective ratings and smoking behavior compared to one’s usual branded cigarettes. We further hypothesized that a graphic (vs. text) warning label affixed to plain packaging would elicit stronger effects, such as fewer favorable subjective ratings, than when it was affixed to commercial packaging.

## Methods

### Design overview

Adults who smoked Marlboro non-menthol cigarettes daily participated in a 50-day, 2 × 3 mixed factorial design (eFigure 1) laboratory-based randomized trial of cigarette package color and warning label effects (ClinicalTrials.gov Identifier: NCT02301351; 25/11/2014). Participants smoked their own preferred brand cigarettes during baseline (5-days) and then completed three consecutive experimental periods (15-days each) where they were randomly assigned to use study cigarettes, provided free-of-charge, with a between-subject warning label manipulation (text-only [cigarettes cause fatal lung disease] vs. graphic [image of diseased lung plus text descriptor]) and within-subject counterbalanced pack color manipulation (within-subject: red, gold, plain). Red and gold packs utilized commercial packaging, while plain packs were created using exact paper weight, color, gloss, and template as commercial packaging but had all marketing materials removed. Participants, but not staff, were blinded by the fact that the cigarettes inside the experimental study packs were their own preferred Marlboro variety. Participants were debriefed about this deception at the end of the final visit. Participants received $500 for completing all procedures during eleven, 2-hour in-person laboratory sessions that occurred every 5 days. The University of Pennsylvania’s Institutional Review Board approved all procedures.

### Participants

We recruited daily, non-treatment seeking, non-menthol Marlboro Red and Gold smokers from the greater Philadelphia area from October 2014 to February 2019 using print and digital media advertisements and contacting previous study participants.

Eligible participants were men and women aged 21–60 who: reported smoking ≥ 5 non-menthol Marlboro Red or Gold cigarettes per day (CPD) for the past 12 months; were not currently undergoing cessation treatment or planning to quit smoking; could communicate fluently in English and provide written informed consent; and planned to remain in the greater Philadelphia area for the study duration. Participants were excluded if they reported using any nicotine product other than cigarettes; consuming ≥ 25 alcohol-containing drinks per week; had a history of substance abuse or serious or unstable disease in the past 12 months; had a history or current diagnosis of chronic obstructive pulmonary disease, stroke, myocardial infarction, psychosis, depression, bipolar disorder, mania, or schizophrenia; were colorblind, pregnant, or breastfeeding; or provided an initial carbon monoxide (CO) assessment < 5 ppm (to verify smoking status); [19] or had breath alcohol concentration (BrAC) reading > 0 at their first session.

### Procedures

Staff scheduled telephone-eligible participants for an initial in-person laboratory visit (i.e., Day 0) to learn more about the study, provide written informed consent, and confirm eligibility. Participants visually displayed their preferred brand to verify their status as a Marlboro Gold or Red smoker, provided an initial CO assessment (Vitalograph Inc., Lenexa, KS) to verify smoking status, and provided a BrAC reading to ensure that they were not under the influence of alcohol. They next completed paper demographic, smoking history, nicotine dependence, and risk perception questionnaires. Participants then smoked two of their preferred brand cigarettes, with 45 min between each. Participants provided CO assessments before and after each cigarette and completed a subjective rating questionnaire after each cigarette. At the end of the session, participants reviewed with staff their future scheduled visits, received date-marked plastic bags, and were instructed on how to collect and store spent filters.

Participants completed in-person sessions every 5 ± 1 days. Session start-times varied by no more than 1 h within-participant to control for diurnal variation in smoking behaviors. Subsequent sessions followed identical procedures, excluding consent and eligibility processes.

### Study cigarettes and packaging manipulations

Participants were randomized to warning label type and pack color order at the second laboratory session and provided their assigned experimental cigarette packs for the duration of the study (eFigure 1). An electronic database randomized participants to warning label (text vs. graphic) and color order (e.g., red, gold, plain; counterbalanced across participants) conditions using a 1:1 allocation ratio, stratified by sex, preferred brand flavor (i.e., red vs. gold), and nicotine dependence. Pack color switches occurred before smoking the second laboratory cigarette on Days 5, 20, and 35.

### Measures

#### Daily cigarette consumption

Smoking behavior was captured via self-reported cigarette consumption for each study day, corroborated at each visit using timeline follow-back procedures, and verified through the collection of spent filters and unused cigarettes [20–23].

#### Subjective cigarette ratings

A 14-item, 100-millimeter visual analog scale [20–22] assessed subjective responses to all cigarettes smoked during sessions. Following previous factor analytic work, [24] three composite subscales were constructed by averaging the individual items in each of three categories: *Product harshness evaluation* (Harshness; Heat; Not mild taste; α = 0.60), *smoking satisfaction* (Very strong; Easy draw; Satisfaction; Not too mild; Not stale; Smoke strength; α = 0.80) and *sensory experience* (Good taste; Good aftertaste; Pleasant smell; α = 0.74).

#### Cigarette risk beliefs

To assess smokers’ beliefs on cigarette harm, participants evaluated eight statements about their current cigarettes relative to “regular” cigarettes (‘are lower in nicotine’; ‘are lower in tar’; ‘are less addictive’; ‘are less likely to cause cancer’; ‘have fewer chemicals’; ‘are healthier’; ‘make smoking safer’; and ‘help people quit smoking’) at the beginning and end of each period on a 5-point response scale (1=’Definitely not true’, 2=’Not true’, 3=’Unsure’, 4=‘True’, 5=’Definitely true’) [25–28]. After examining the item’s distributional properties (eFigure 2), we subsequently scored the ‘definitely not true’ and ‘not true’ responses as 0 = ‘factually correct’, and all others as 1 = ‘uncertain or incorrect’. A risk belief composite score indicating the cumulative number of ‘incorrect or uncertain’ cigarette risk beliefs was also constructed (range: 0–8).

### Covariates

Demographic and smoking history information measures were assessed at the first study visit, including: age, sex, race/ethnicity, nicotine dependence (assessed using the Fagerström Test for Nicotine Dependence) [29].

### Analytic plan

Sample characteristics were compared for text-only warning and graphic warning groups and for study completers versus non-completers. Categorical and continuous variables were analyzed using χ^2^ tests of independence and Welch two sample T-tests, respectively.

To leverage the full power of our design and account for sample size differences in pack color preferences (e.g., sample majority smoked Marlboro Red cigarettes), we coded the experimental pack color condition to be congruent when the smokers’ preferred pack color matched the experimental period (e.g., red pack preference in the red pack period) and incongruent when the pack colors were misaligned (e.g., red pack preference in the gold pack period). The coding of plain pack color condition was unchanged.

We employed conditional mixed-effects models with 95% confidence intervals using the “lme4” [30] and “lmerTest” [31, 32] packages in R [33] with multivariate *t* adjusted post-hoc tests [34] using the “emmeans” package [35, 36] to examine between and within group differences. Likelihood ratio testing determined random intercept models to best fit the data. Models testing binary outcomes (i.e., risk beliefs) utilized binomial distributions, while the model testing the composite count outcome (i.e., summated risk beliefs) utilized a Poisson distribution. Continuous outcomes (i.e., daily cigarette consumption and subjective cigarette ratings) utilized Gaussian distributions. For each continuous outcome, data were averaged across the 5-day baseline and 15-day experimental periods. Before data aggregation, baseline adjusted conditional growth models with estimated marginal means of linear trends were used to confirm that the slope parameters within each experimental period did not significantly differ from zero. All mixed-effects models utilized a bound optimization by quadratic approximation (BOBYQA) with a set maximum of 200,000 iterations [37]. Binary outcome models included a warning label by experimental block by time interaction term, while continuous outcomes included the warning label by experimental block interaction. All models adjusted for pack color preference, experimental pack color randomization order, age, sex, race/ethnicity, education, and nicotine dependence. Continuous covariates were standardized (Mean = 0; SD = 1). All tests were 2-sided and used a priori significance of *p* < .05.

## Results

**Fig. 1:**
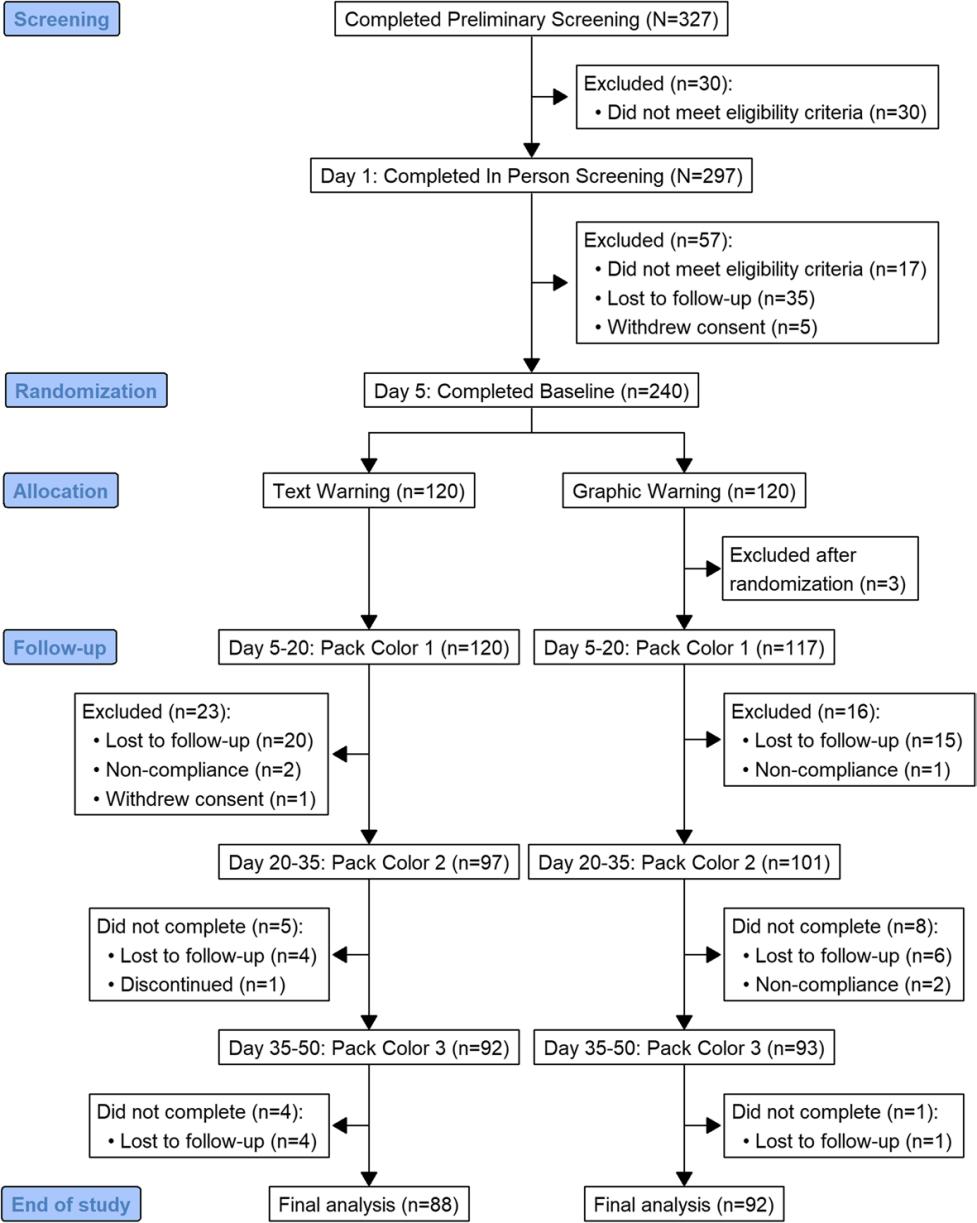
CONSORT flow diagram depicting study recruitment and retention

A total of 327 participants completed the preliminary screening and attended the baseline visit, of which 30 did not meet in-person eligibility criteria (Fig. 1). Of the 297 eligible participants who signed an informed consent, 17 did not meet adherence criteria, 35 were lost to follow-up, and 5 withdrew consent leaving 240 participants (120 per warning group) to achieve randomization. Less than a quarter of participants (N = 57) did not complete the full 50-day trial and 3 were excluded post-randomization (participants enrolled prior to trial registration) leaving 180 participants in the analytic sample. No differences in sample characteristics were observed between warning label groups (Table 1) or by attrition.

**Table 1:** Sample characteristics by study randomization to warning label condition

		Warning Label Condition		
	Overall	Text	Graphic	
Characteristic	(*N* = 180)	(*N* = 88)	(*N* = 92)	*P*-value
Age in years, Mean (SD)	42.98 (10.93)	43.62 (11.05)	42.37 (10.84)	0.44 ^a^
Sex, n (%)				0.57 ^b^
Male	119 (66.1%)	60 (68.2%)	59 (64.1%)	
Female	61 (33.9%)	28 (31.8%)	33 (35.9%)	
Race/Ethnicity, n (%)				0.30 ^b^
White	125 (69.4%)	57 (64.8%)	68 (73.9%)	
Black	31 (17.2%)	16 (18.2%)	15 (16.3%)	
Other	24 (13.3%)	15 (17.0%)	9 (9.8%)	
Education, n (%)				0.78 ^b^
High school or less	71 (39.4%)	33 (37.5%)	38 (41.3%)	
Some college	77 (42.8%)	40 (45.5%)	37 (40.2%)	
College grad or beyond	32 (17.8%)	15 (17.0%)	17 (18.5%)	
Cigarette flavor preference, n (%)				0.36 ^b^
Red	140 (77.8%)	71 (80.7%)	69 (75.0%)	
Gold	40 (22.2%)	17 (19.3%)	23 (25.0%)	
Past 7-day daily cigarette consumption, Mean (SD)	16.16 (7.27)	15.73 (6.94)	16.57 (7.59)	0.44 ^a^
Nicotine dependence, Mean (SD)	5.31 (2.16)	5.56 (2.10)	5.07 (2.19)	0.13 ^a^

^a^Calculated using Welch Two Sample T-test. ^b^ Calculated using the χ2 test

### Change in cigarette consumption

During the 5-day baseline period of ad-lib smoking, participants consumed an average of 13.1 cigarettes per day (CPD; SE = 0.86) for those randomized to the text-only warning and 14.1 CPD (SE = 0.86) for those randomized to the graphic warning (Table 2). Over the course of the trial, self-reported daily cigarette consumption was highly correlated with the number of retuned, used, cigarette filters (within-subjects repeated measures correlation = 0.84 [95%CI = 0.83, 0.85], *p* < .001).Compared to baseline, participants smoked an increased number of cigarettes during each experimental pack color condition regardless of their warning label group assignment. Cigarette consumption within the text-only warning group increased from baseline by more than an average of 3 CPD (B’s range = 3.16–3.59), whereas consumption within the graphic warning group increased by just over an average of 2.50 CPD (B’s range = 2.64–2.88). No significant differences in cigarette consumption were observed between the warning label groups during either the baseline or experimental periods, nor were there any differences between the congruent, incongruent, and plain package periods.

**Table 2:** Post-hoc pairwise multiple comparisons of change in daily cigarette consumption, subjective cigarette ratings, and risk belief uncertainty using adjusted between and within subject contrasts

Smoking Outcome	Subject Contrast	Warning Type	Baseline		Experimental Pack Color Condition		
			Usual		Congruent	Incongruent	Plain
Consumption^1^	Between	Text	*Ref*		*Ref*	*Ref*	*Ref*
		Graphic	0.99 (-0.93, 2.90)		0.28 (-1.64, 2.20)	0.57 (-1.35, 2.48)	0.32 (-1.60, 2.24)
	Within	Text	*Ref*		3.59 ( 2.85, 4.33)^***^	3.16 ( 2.41, 3.90)^***^	3.30 ( 2.56, 4.05)^***^
		Graphic	*Ref*		2.88 ( 2.16, 3.61)^***^	2.74 ( 2.10, 3.46)^***^	2.64 ( 1.92, 3.36)^***^
Harshness^1^	Between	Text	*Ref*		*Ref*	*Ref*	*Ref*
		Graphic	1.13 (-1.85, 4.10)		-1.43 (-4.42, 1.56)	-2.66 (-5.64, 0.32)	-1.36 (-4.35, 1.62)
	Within	Text	*Ref*		1.21 (-1.40, 3.82)	1.68 (-0.93, 4.28)	1.80 (-0.81, 4.41)
		Graphic	*Ref*		-1.35 (-3.91, 1.22)	-2.11 (-4.65, 0.43)	-0.69 (-3.24, 1.86)
Satisfaction^1^	Between	Text	*Ref*		*Ref*	*Ref*	*Ref*
		Graphic	-0.48 (-4.32, 3.37)		-0.16 (-4.02, 3.70)	-0.12 (-3.97, 3.73)	-0.30 (-4.15, 3.56)
	Within	Text	*Ref*		-0.25 (-3.31, 2.77)	-2.19 (-5.24, 0.85)	-1.44 (-4.49, 1.60)
		Graphic	*Ref*		0.05 (-2.94, 3.03)	-1.84 (-4.80, 1.13)	-1.27 (-4.24, 1.71)
Sensory experience^1^	Between	Text	*Ref*		*Ref*	*Ref*	*Ref*
		Graphic	1.90 (-4.14, 7.94)		1.52 (-4.35, 7.57)	1.59 (-4.45, 7.64)	-0.10 (-6.15, 5.94)
	Within	Text	*Ref*		-0.10 (-3.41, 3.22)	-1.53 (-4.85, 1.79)	-0.41 (-3.73, 2.91)
		Graphic	*Ref*		-0.47 (-3.73, 2.78)	-1.83 (-5.06, 1.40)	-2.41 (-5.65, 0.83)
Risk belief	Between	Text	*Ref*		*Ref*	*Ref*	*Ref*
composite count^2,3^		Graphic	0.96 (0.70, 1.32)		0.74 (0.54, 0.99)^*^	0.72 (0.53, 0.97)^*^	0.77 (0.57, 1.04)
	Within	Text	*Ref*		1.70 (1.43, 2.03)^***^	2.01 (1.70, 2.38)^***^	2.16 (1.82, 2.55)^***^
		Graphic	*Ref*		1.30 (1.09, 1.55)^***^	1.51 (1.27, 1.78)^***^	1.73 (1.46, 2.04)^***^

All models adjusted for pack color preference, experimental pack color randomization order, age, sex, race/ethnicity, education, and nicotine dependence. ^1^From random intercept gaussian conditional mixed-effects models using multivariate *t* adjusted post-hoc tests with separate models run to examine differences between warning groups and within-subject change from baseline. Data expressed as Odds Ratios (95% CIs).

^2^From random intercept Poisson conditional mixed-effects models using multivariate *t* adjusted post-hoc tests with separate models run to examine differences between warning groups and within-subject change from baseline. Data expressed as Incidence Rate Ratios (95% CIs).

^3^Cumulative counts of incorrect or uncertain cigarette risk beliefs

* *p* < .05; ** *p* < .01; *** *p* < .001

### Change in subjective cigarette ratings

On average, participants rated their usual cigarettes as satisfying (VAS mean = 65.1 [95%CI = 62.4, 67.7]), providing a good sensory experience (VAS mean = 55.7 [95%CI = 51.4, 60.0]) and not being overly harsh (VAS mean = 51.7 [95%CI = 49.7, 53.7]). No differences in subjective cigarette ratings were observed between the pack warning groups, nor was any within-subject change observed during any experimental condition relative to baseline (Table 2).

### Change in cigarette risk beliefs

**Fig. 2:**
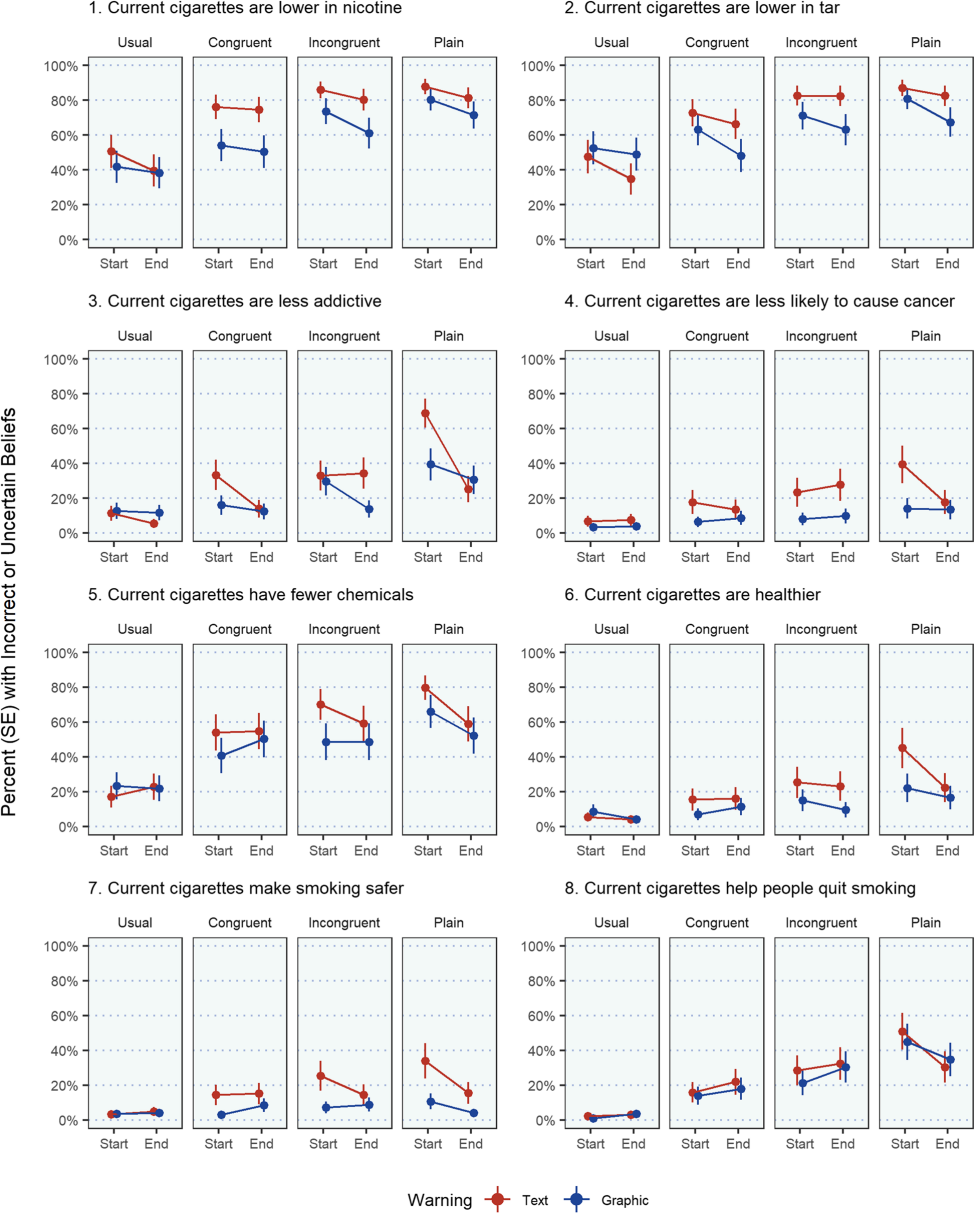
Change in cigarette incorrect or uncertain risk beliefs across cigarette pack color use periods by warning label group

During baseline, participants predominately held factually correct risk perceptions of their own cigarettes (Mean = 6.02, SE = 0.17) and very few incorrect responses (Mean = 0.31, SE = 0.05). Visual examination of the eight risk belief items over time revealed that anything other than correct perceptions were largely driven by uncertainty and not incorrect beliefs. Further, the proportion holding incorrect beliefs did not increase over time (eFigure 2), and risk beliefs did not change during baseline (Fig. 2). Baseline uncertainty was highest for statements surrounding tar (33.9-38.3%), nicotine (31.7-33.9%), and chemical content (29.0-29.5%) and lowest for statements around the likelihood of causing cancer (13.1-16.4%), safety (10.4-11.4%), and helpfulness for quitting (7.1-10.4%).

Exposure to the text-only warning increased the baseline rate of 1.48 (SE = 0.21) uncertain or incorrect beliefs to 2.52 (SE = 0.34; IRR = 1.70 [95%CI = 1.43, 2.03]) when the experimental pack color matched the smokers preference (i.e., congruent), to 2.98 (SE = 0.40; IRR = 2.01 [95%CI = 1.70, 2.38]) when pack color did not match the preference (i.e., incongruent), and to 3.19 (SE = 0.43; IRR = 2.16 [95%CI = 1.82, 2.55]) when the pack was plain (Table 2). Similarly, exposure to the graphic warning increased the baseline rate of 1.42 (SE = 0.20) uncertainties to 1.84 (SE = 0.25; IRR = 1.30 [95%CI = 1.09, 1.55]) with congruent color packs, to 2.14 (SE = 0.29; IRR = 1.51 [95%CI = 1.27, 1.78]) with incongruent color packs, and to 2.45 (SE = 0.33; IRR = 1.73 [95%CI = 1.46, 2.04]) with plain packs. Consequently, compared to the text-only group, those in the graphic group experienced less of an increase in uncertainty when their packs were congruent (IRR = 0.73 [95%CI = 0.54, 0.99]) or incongruent (IRR = 0.72 [95%CI = 0.53, 0.97]) in color, but not when they were plain (eFigure 3).

Within the text-only warning condition, the odds of reporting an incorrect or uncertain risk belief increased from baseline for all eight risk belief items regardless of the experimental pack color condition (ORs range:2.39–25.55; Table 3). Within the graphic warning condition, the odds of incorrect or uncertain risk beliefs increased from baseline for all but the ‘safer’ risk belief item, but only when the packaging color was plain (ORs range:2.40-25.69). When the graphic warning group’s pack color was incongruent or congruent, the odds of reporting an incorrect or uncertain risk belief increased from baseline only for statements about ‘fewer chemicals’ and ‘help with quitting’ (ORs range:2.87–17.82). Between warning conditions, packs with graphic warnings, as compared to text-only warnings, reduced uncertainty about study packs being ‘safer’ than their usual pack, an effect present across all experimental conditions (ORs range:0.23–0.36).

**Table 3:** Post-hoc pairwise multiple comparisons of change in incorrect or uncertain risk beliefs using adjusted between and within subject contrasts

Risk Belief Outcome^1^	Subject Contrast	Warning Type	Baseline	Experimental Pack Color Condition		
			Usual	Congruent	Incongruent	Plain
Lower nicotine	Between	Text	*Ref*	*Ref*	*Ref*	*Ref*
		Graphic	0.81 (0.38, 1.76)	0.36 (0.16, 0.77)^**^	0.42 (0.19, 0.92)^*^	0.57 (0.26, 1.26)
	Within	Text	*Ref*	3.74 (1.83, 7.68)^***^	6.08 (2.89, 12.78)^***^	6.85 (3.23, 14.52)^***^
		Graphic	*Ref*	1.65 (0.82, 3.29)	3.14 (1.55, 6.35)^***^	4.81 (2.33, 9.92)^***^
Lower tar	Between	Text	*Ref*	*Ref*	*Ref*	*Ref*
		Graphic	1.49 (0.68, 3.25)	0.55 (0.25, 1.20)	0.43 (0.19, 0.96)^*^	0.52 (0.23, 1.18)
	Within	Text	*Ref*	3.31 (1.61, 6.82)^***^	6.82 (3.20, 14.55)^***^	8.14 (3.77, 17.58)^***^
		Graphic	*Ref*	1.22 (0.61, 2.45)	1.99 (0.99, 4.00)	2.87 (1.41, 5.86)^***^
Less addictive	Between	Text	*Ref*	*Ref*	*Ref*	*Ref*
		Graphic	1.60 (0.67, 3.81)	0.58 (0.25, 1.31)	0.51 (0.23, 1.13)	0.62 (0.28, 1.36)
	Within	Text	*Ref*	3.29 (1.44, 7.50)^**^	5.87 (2.58, 13.33)^***^	9.99 (4.30, 23.22)^***^
		Graphic	*Ref*	1.18 (0.54, 2.59)	1.87 (0.87, 4.04)	3.86 (1.80, 8.26)^***^
Less cancerous	Between	Text	*Ref*	*Ref*	*Ref*	*Ref*
		Graphic	0.48 (0.17, 1.36)	0.44 (0.16, 1.16)	0.28 (0.11, 0.74)^*^	0.43 (0.17, 1.09)
	Within	Text	*Ref*	2.40 (1.05, 5.47)^*^	4.44 (1.94, 10.15)^***^	4.87 (2.13, 11.17)^***^
		Graphic	*Ref*	2.17 (0.84, 5.60)	2.61 (1.02, 6.68)^*^	4.30 (1.68, 10.97)^***^
Fewer chemicals	Between	Text	*Ref*	*Ref*	*Ref*	*Ref*
		Graphic	1.18 (0.49, 2.84)	0.70 (0.30, 1.65)	0.51 (0.22, 1.21)	0.61 (0.26, 1.45)
	Within	Text	*Ref*	4.84 (2.24, 10.44)^***^	7.47 (3.40, 16.40)^***^	9.65 (4.32, 21.56)^***^
		Graphic	*Ref*	2.87 (1.35, 6.08)^**^	3.25 (1.53, 6.89)^***^	4.99 (2.31, 10.78)^***^
Healthier	Between	Text	*Ref*	*Ref*	*Ref*	*Ref*
		Graphic	1.30 (0.46, 3.72)	0.52 (0.19, 1.41)	0.43 (0.16, 1.13)	0.49 (0.19, 1.28)
	Within	Text	*Ref*	3.77 (1.58, 9.04)^***^	6.45 (2.67, 15.54)^***^	9.76 (3.99, 23.86)^***^
		Graphic	*Ref*	1.52 (0.62, 3.72)	2.12 (0.88, 5.13)	3.69 (1.54, 8.88)^***^
Safer	Between	Text	*Ref*	*Ref*	*Ref*	*Ref*
		Graphic	0.94 (0.33, 2.66)	0.32 (0.12, 0.84)^*^	0.36 (0.14, 0.94)^*^	0.23 (0.09, 0.60)^**^
	Within	Text	*Ref*	4.07 (1.67, 9.92)^***^	5.64 (2.31, 13.74)^***^	7.25 (2.96, 17.77)^***^
		Graphic	*Ref*	1.37 (0.53, 3.51)	2.18 (0.88, 5.39)	1.79 (0.71, 4.51)
Help with quitting	Between	Text	*Ref*	*Ref*	*Ref*	*Ref*
		Graphic	0.74 (0.24, 2.21)	0.82 (0.34, 1.95)	0.79 (0.34, 1.84)	0.98 (0.42, 2.28)
	Within	Text	*Ref*	8.79 (3.35, 23.04)^***^	16.68 (6.29, 44.25)^***^	25.69 (9.52, 69.34)^***^
		Graphic	*Ref*	9.73 (3.53, 26.83)^***^	17.82 (6.38, 49.73)^***^	34.16 (11.92, 97.90)^***^

*Note. All models adjusted for pack color preference, experimental pack color randomization order, age, sex, race/ethnicity, education, and nicotine dependence. ^1^From random intercept binomial conditional mixed-effects models using multivariate *t* adjusted post-hoc tests with separate models run to examine differences between warning groups and within-subject change from baseline. Data expressed as Odds Ratios (95% CIs) and represent the increased odds of incorrect or uncertain beliefs relative to correct beliefs averaged over time for the respective experimental period

* *p* < .05; ** *p* < .01; *** *p* < .001

## Discussion

In this randomized trial, we examined the effects of warning labels and cigarette package colors on risk beliefs, subjective ratings, and smoking behavior. When we experimentally manipulated the cigarette packaging in the form of two potential tobacco regulatory policies, warning labels and pack color, we observed an increase in uncertainty around several key risk beliefs related to smoking. These uncertainties occurred despite maintaining the participants’ preferred cigarette brand and variant. Notably, the observed increase did not appear to be driven by an increase in incorrect beliefs but instead by the generation of new uncertainty in what were previously correct beliefs held by most of the sample. Despite the overall increase in uncertainty, individuals randomized to receive a graphic warning had a 25% lower increase in uncertainty than those in the text-only group, but only when the warning was placed on a commercial pack (i.e., red or gold). Thus, package color appears to increase uncertainty about the associated health risk, but graphic warnings may attenuate this affect. Nevertheless, when these two policies are introduced to current smokers, they have increased uncertainty, but not incorrectness, about product risk. This is an important distinction. These results demonstrate the need for supportive education campaigns timed with policy implementation to reduce uncertainties, rather than correctives which would better counteract incorrect knowledge.

Graphic warning labels and plain packaging have been adopted in many countries with varying levels of impact on public health [3]. Our study is the first to examine the impact of graphic warning labels, plain packaging, and package color modification on cigarette consumption, subjective ratings, and risk perceptions in US smokers. However, we found no change in subjective ratings or reduced cigarette consumption, an effect consistent with a recent US trial examining plain packaging with graphic warnings  [7, 38, 39]. Yet, the results of this trial have broad implications on how to investigate the impact of policy, as policies are rarely enacted in isolation [40]. For example, the implementation of graphic warnings has been accompanied by tax increases, [41, 42] smoke-free air laws, [43–45] and bans on tobacco advertisements [43, 46, 47]. Further, the recommended WHO Framework Convention on Tobacco Control policies have not received global adoption, with many countries still working on their implementation. Meanwhile, new or strengthened policies are being considered across the globe, such as reduced nicotine standards, [48, 49] flavor bans, [50, 51] as well as plain packaging, [52] and these may or may not be introduced alone or as a series of steps to improve public health. Our study demonstrates the importance of examining the impact of multiple policies and exploring what type of effects occur among outcomes of interest. For instance, policies that intend to decrease initiation and smoking prevalence, [4] increase knowledge, [6] or make cigarettes less appealing [52] may unintentionally generate uncertainty in risk. However, this uncertainty can be mitigated if decision-makers are aware of the type of risk error. Our study supports the need to examine multiple policies simultaneously, which is likely to more closely resemble the milieu in which the policies will be implemented.

This study supports the importance of capturing uncertainty in risk belief assessments [53]. Often, dedicated questions about uncertainty are not included in risk appraisals but are sometimes included as response options. When this occurs, the level may often be dropped or combined into a false belief category [15]. Our results indicate that when smokers have misperceptions about cigarette risks, it is because they are largely uncertain rather than misinformed and that policies that provide brief health information may exacerbate this uncertainty. Additional health information campaigns may be necessary, if not vital, to clearly convey correct information that the public comprehends to update beliefs after such policies are enacted successfully. These campaigns would benefit from strategies designed to inform smokers of the risk as a means to correct uncertainty, instead of using corrective messaging to counter incorrect beliefs. Identifying which beliefs smokers are uncertain about is valuable because uninformed individuals are more likely to update their beliefs than misinformed individuals after exposure to corrective information [54]. It is possible that the uncertainty generated in this study indicates ambiguity or the reluctance to think carefully about the risks, [53] extant research suggests these responses are meaningful and reflect actual uncertainty [55]. Nevertheless, even if smokers are non-committal to a belief, they are unlikely to differ from uninformed smokers regarding belief updating [54].

This study should be considered in light of its strengths and limitations. First, participants were provided with free cigarettes, which does not reflect how smokers obtain these products and likely contributed to the increased cigarette consumption [56, 57]. Free cigarettes have been shown to increase cigarette use by roughly 4.4 CPD, 59 an effect consistent with our observations which suggests that the increased cigarette consumption we report is likely not the result of the examined policies. Comparing the results of the graphic warning group to the text-only warning group further supports this consideration. Second, we used a graphic and text warning which conveyed risk information about lung disease, a smoking-related outcome that most Americans are aware of and believe to be true, [58] which likely limited the impact on altering risks beliefs and smoking behavior. Given FDA’s recent strategy to focus on lesser-known risks with a new set of warning images, we might expect these new labels to have less of an impact on uncertainty and increase understanding of the risks. Third, the number of incorrect risk beliefs were few which limited our ability to analytically examine changes in misperceptions over time. We chose to combine incorrect and uncertain risk beliefs into a single category as opposed to omitting the incorrect responses which would have reduced the sample size and led to differential missingness in risk belief responses over time. Finally, the study was comprised of adult, daily, Marlboro non-menthol smokers who preferred one of two brand variants and held no intentions to quit smoking, thus limiting the generalizability of the study’s findings. Strengths of the study include using text and graphic warning label conditions, and having packs created to test incongruent colors and plain packaging. We also elected to include a ‘Unsure’ response to cigarette risk belief items which allowed participants to provide responses that better match the cognitive processes used in everyday life to form risk perceptions [53].

## Conclusion

Pack color modification may generate increased uncertainty about several key cigarette risk beliefs, though graphic warnings may attenuate these effects. These results demonstrate the importance of providing education to reduce uncertainty about the risks of cigarette products when enacting regulatory policies affecting their packaging.

### Supplementary Information

**Additional file 1: eFigure 1.** Overview of study design and procedures. **eFigure2.** Risk Belief Endorsement across Cigarette Pack Color Use Periods by Warning Label Group. **eFigure 3.** Change in Cumulative Count of Cigarette Incorrect or Uncertain Risk Beliefs across Baseline and Experimental Cigarette Pack Color Periods by Warning Label Group (N=183)

## Data Availability

Data are available upon reasonable request by contacting strasse3@pennmedicine.upenn.edu. [Corresponding author]
