# Supplementary material for: Effects of cigarette package colors and warning labels on marlboro smokers’ risk beliefs, product appraisals, and smoking behavior: a randomized trial
Source: BMC Public Health. 2023 Oct 27;23:2111. doi: 10.1186/s12889-023-17024-5 (PMC10605973; doi:10.1186/s12889-023-17024-5)
Supplement: Supplementary file 1 — Additional file 1: eFigure 1. Overview of study design and procedures. eFigure2. Risk Belief Endorsement across Cigarette Pack Color Use Periods by Warning Label Group. eFigure 3. Change in Cumulative Count of Cigarette Incorrect or Uncertain Risk Beliefs across Baseline and Experimental Cigarette Pack Color Periods by Warning Label Group (N=183) [file 12889_2023_17024_MOESM1_ESM.docx]

**Supplemental Materials**

**
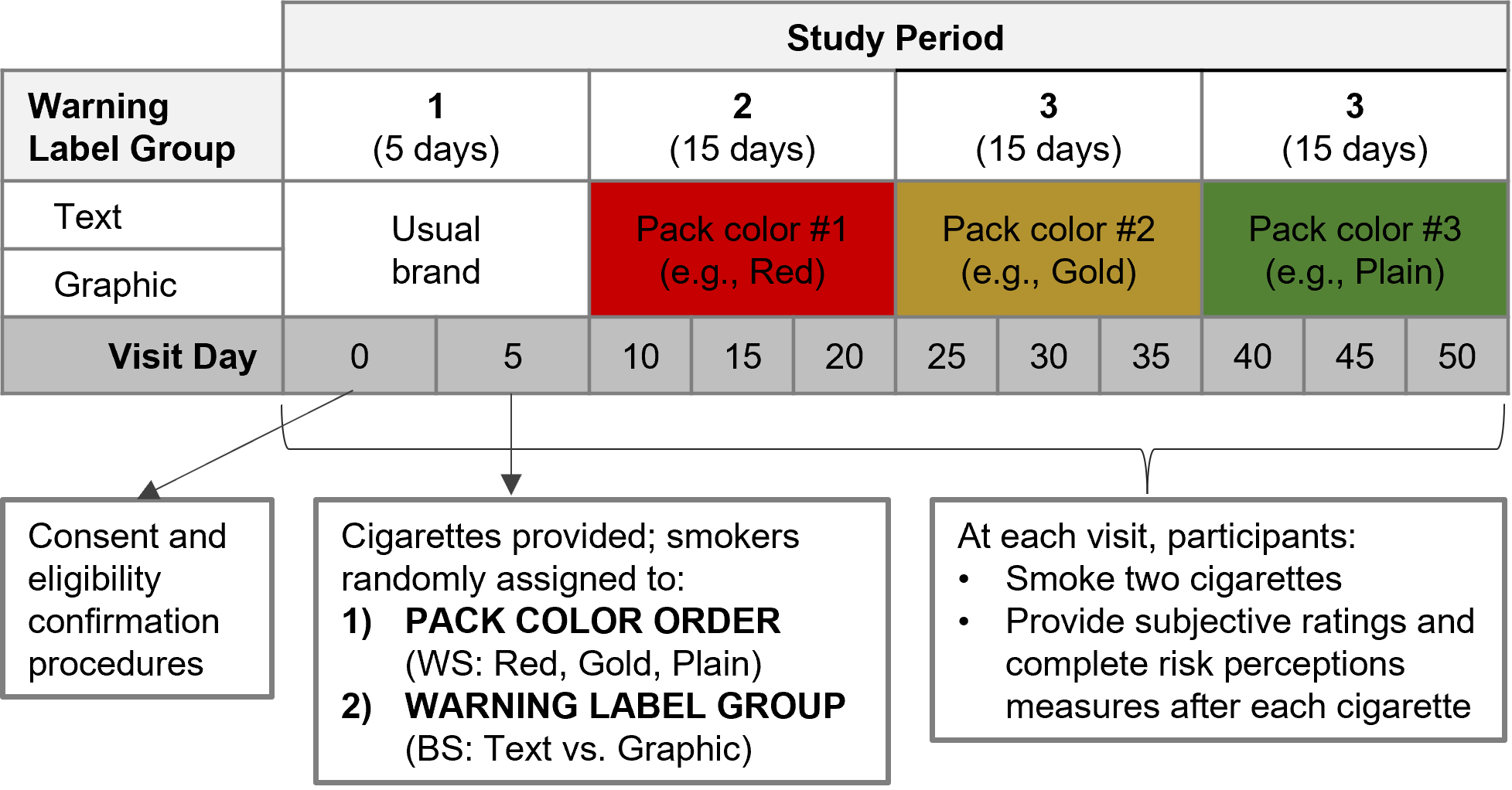
eFigure 1.** Overview of study design and procedures.

**
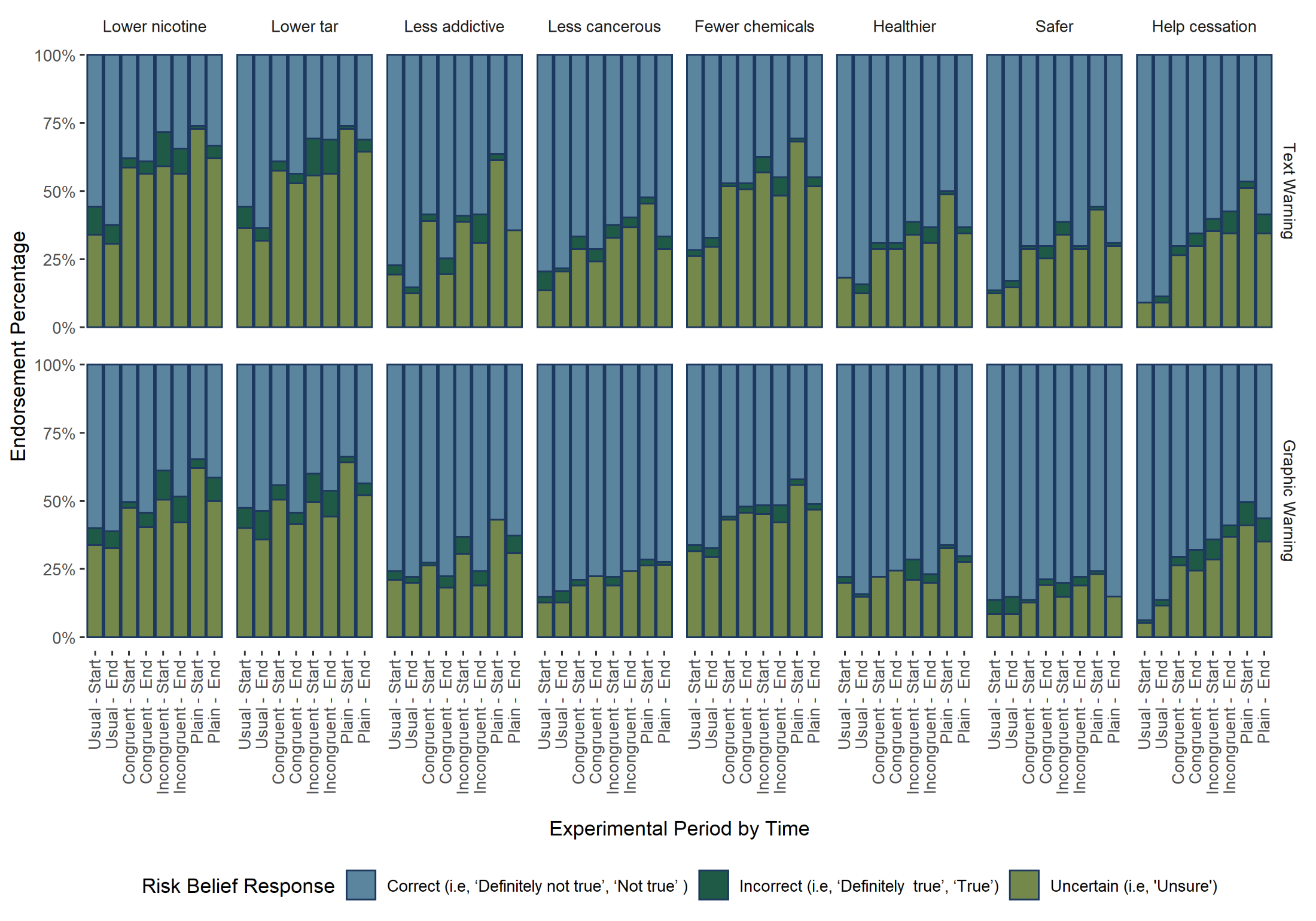
 eFigure** **2.** Risk Belief Endorsement across Cigarette Pack Color Use Periods by Warning Label Group

**
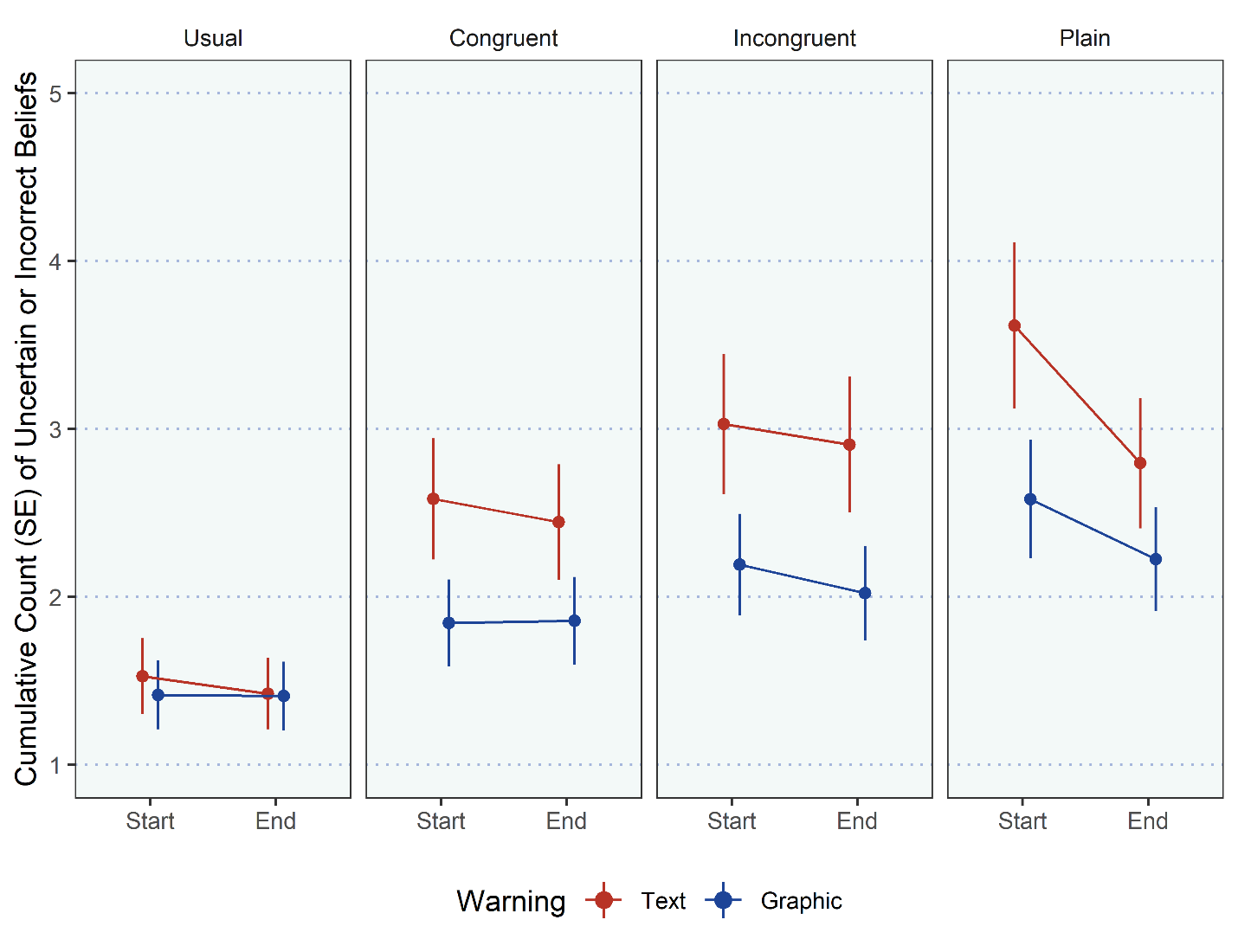
eFigure 3.** Change in Cumulative Count of Cigarette Incorrect or Uncertain Risk Beliefs across Baseline and Experimental Cigarette Pack Color Periods by Warning Label Group (N=183)
